# Evidence for interventions to promote mental health and reduce stigma in Black faith communities: systematic review

**DOI:** 10.1007/s00127-021-02068-y

**Published:** 2021-04-18

**Authors:** Louisa Codjoe, Sarah Barber, Shalini Ahuja, Graham Thornicroft, Claire Henderson, Heidi Lempp, Joelyn N’Danga-Koroma

**Affiliations:** 1 Health Service and Population Research Department, Institute of Psychiatry, Psychology and Neuroscience (IoPPN), King’s College London, De Crespigny Park, London, SE5 8AF UK; 2 Centre for Implementation Science and Centre for Global Mental Health, Institute of Psychiatry, Psychology and Neuroscience (IoPPN), King’s College London, De Crespigny Park, London, SE5 8AF UK; 3 Centre for Rheumatic Diseases, Department of Inflammation Biology, King’s College London, Weston Education, 10, Cutcombe Rd, London, SE5 9RJ UK

**Keywords:** Black community, Faith community, Intervention, Mental Health, Stigma

## Abstract

**Purpose:**

There are significant documented inequalities for the Black community in the UK in relation to mental health care. Research has also indicated that cultural difference exists in pathways into, and engagement with, mental health services. To reduce inequalities and improve engagement with mental health services, it is important that professionals utilise culturally appropriate community networks to increase mental health awareness and reduce stigma. This systematic review considers research in Black faith settings, with two linked aims to review the evidence for the effectiveness of (i) mental health interventions, and (ii) other health stigma interventions as the latter have been implemented in Black faith settings. The review identified ‘active ingredients’ of interventions for this population that can be applied in future work. The authors seek to draw from the mental health and wider health stigma literature to inform the design of the ON TRAC project, a collaborative partnership between King’s College London, South London and Maudsley NHS Foundation Trust and Black faith community groups in Southwark and Lambeth, London, in this currently under-researched area.

**Methods:**

A systematic search of ten major medical and social sciences databases was conducted in 2019, for studies on mental health or other health stigma interventions in Black faith settings. PRISMA guidelines were followed and search terms and search strategy ensured all possible studies were identified for review.

**Results:**

The review identified sixteen studies for inclusion. Ten were quantitative studies, four qualitative studies and two systematic reviews. Active ingredients of interventions included utilisation of ‘bottom up’ development of approaches and mental health champions. Multiple factors were found to influence effective implementation. Co-production and partnership working are key to ensure that an acceptable and accessible intervention is agreed.

**Conclusion:**

Evidence for the effectiveness of interventions focused on mental health awareness and stigma reduction in the Black faith community is limited due to the low quality of studies. This review sheds light on the lessons learnt and necessary key requirements for interventions that can guide future projects.

**Study registration**: PROSPERO registration number: CRD42018110068

**Supplementary Information:**

The online version contains supplementary material available at 10.1007/s00127-021-02068-y.

## Introduction

There are significant documented inequalities for the Black community in relation to mental health care in the UK [2]. An extensive literature has been published on this topic and seeks to explain this disparity [49], but no single explanation has been identified. The literature indicates, however, that pathways into care for Black African and Caribbean communities are more likely to be traumatic and via the police [2], that the incidence of serious mental health conditions, such as psychosis are higher in Black neighbourhoods [47], and that service satisfaction is significantly lower among this group (National Institute for Mental Health in England (NIMHE) [45]). Barnett et al. [5] found that Black African and Caribbean service users had significantly increased odds of being compulsorily admitted to hospital compared with white ethnic groups. Further, Black Caribbean service users were also significantly more likely to be readmitted to hospitals compared with white ethnic groups /counterparts [5]. Such presentations are additionally more likely to be associated with negative clinical, social, and occupational outcomes [41].

The reasons for this disparity have been long debated. Some argue that it is a function of higher rates of serious mental illness in this group [23], which may be driven by social determinants of poor mental health and structural discrimination of minorities outside of the health system. However, determining the seriousness of mental illness involves a risk assessment, which may be impacted by racial prejudice [42]. Others argue that rates in the US are higher due to later presentation to health services [51], but this has not been substantiated in UK populations [22, 40]. In UK Black African and Caribbean populations, it is more likely that entry to mental health services is via the criminal justice system than through primary health care [22]. In addition, the longitudinal trajectory of psychosis in Black service users typically has longer periods of admission and compulsory re-admission [3]. There are other examples of mental health inequalities for Black African and Caribbean groups. People from ethnic minorities are less likely than their White British counterparts to have contacted their general practitioner (GP) about mental health concerns, to be prescribed antidepressants, or to be referred to specialist mental health services [38]. Indeed, a recent report highlighted the underutilisation of services by Black African and Caribbean groups who are hard to reach due to linguistic and cultural barriers (NICE, 2017). Such failures by the professional health services plausibly leads to fear and mistrust in the community, perpetuating a cycle of poor access and increased requirement for coercion [12].

Research also indicated that cultural difference exists in pathways into, and engagement with, mental health services [7]. Some communities may have culturally informed explanations of mental illness and consequently seek ways to manage symptoms, which are perceived as more relevant and accessible [24]. In addition, stigma, and discrimination, both in terms of mental illness stigma and experiences of being stigmatised, have been identified as having a negative effect on help-seeking for mental health difficulties [17]. Recent studies have explored attitudes towards mental health problems in Black faith communities in South London [12, 34, 35]. There is some evidence that communities may view mental illness as a weakness or moral failing, resulting in shame, secrecy, and reluctance to seek help. There is a desire for social distance, which impacts individuals with mental health problems as well as their families. In the current climate, COVID-19 has exposed the extent of these issues and shown it as a determinant of population health (Egede et al. 2020). Some have argued that racism underpins the design of mental health services which demand a Westernised view of health [11]. This results in a failure of the service provider to adequately explore how to accommodate different ways of engaging culturally diverse communities so there is equity in access to mental health care. This review is interested in the significance the Black Church has had in the history of Black African and Black Caribbean people worldwide, in particular as a space to connect with community, seek help, guidance and as a first point of contact when experiencing mental distress [11]. Research findings indicate that in many Black African and Caribbean service users consider a positive relationship with their faith as central to wellness, rather than adopt a medicalised view of care [11].

The UK has seen an exponential growth of Black Majority Churches (BMC) and within a local government area the authors have conducted research that sees over 20,000 people Black African and Caribbean people gathering to worship weekly across 240 BMC’s. This area represents the greatest concentration of African Christianity in the world outside BMC’s in African (University of Roehampton, 2013). Some authors have emphasised the need for service development initiatives to reduce mental illness stigma, improve engagement and consider combined anti-stigma and mental health awareness interventions [56] for mental health providers in Black church congregations [8]. This may include faith-based interventions (e.g., where external agencies e.g., statutory services collaborate with faith communities to develop interventions) or faith-placed interventions (e.g., developed within the faith community). Stigma reduction and mental health awareness interventions, whilst important, are only one variable in a complex picture of well-documented systemic racism and discrimination in mental health service provision. However, to improve access, experience, engagement, and outcomes with statutory services, it is important that healthcare professionals utilise culturally appropriate community networks to increase mental health awareness and develop partnerships [24].

This review examines the evidence for mental health or stigma reduction interventions in Black faith communities. Specific search terms relating to mental health ensured capture of this literature. However, searching for the broader health stigma research in this setting allowed for cross-learning from other health conditions, such as HIV. This is in keeping with the move away from a siloed approach to stigma research, as we come to understand the social ecological and intersectional nature of stigma (Logie [33]). Through the identification and synthesis of relevant quantitative and qualitative studies, this systematic review aims to: (1) review evidence for the effectiveness of interventions to promote mental health or to reduce health stigma in Black faith communities; (2) better understand the active ingredients of mental health and/or health stigma reduction interventions for Black faith communities; and (3) identify factors which influence effective implementation.

## Methods

This systematic review followed a published protocol (Prospero, CRD42018110068) and complies with the preferred reporting items for systematic reviews and meta-analyses (PRISMA) statement (Moher [39]).

### Search strategy

In September 2019, ten major medical and social sciences databases (Medline, ASSIA, BNI, IBSS, Social Policy and Practice, Cochrane Library, CINAHL, SSCI, Global Health and PsycINFO) were searched. A comprehensive search strategy was devised, combining free-text items with subject headings (exploded in databases where possible). Full search terms can be found in Online Appendix A. The search was not limited by year or geographical area but was restricted to English language publications. Two authors (SB, LC) manually searched the reference lists of included studies.

### Study selection, inclusion, and exclusion criteria

The impetus for this review was to examine evidence to inform the design of a local collaboration between Black faith communities and mental health services in South London. Preliminary searches indicated that to meet the aims of reviewing the effectiveness of (i) mental health interventions; and (ii) health stigma interventions in Black faith communities, the inclusion criteria would need to differ. For aim (i) studies were included if they evaluated a mental health awareness intervention within Black faith communities. However, for aim (ii), given the paucity of literature on stigma reduction interventions in the Black community, studies were included using a broader inclusion criteria and included stigma reduction interventions focused on all areas of health. Studies were included if they were eligible for evaluation using the and Integrated quality Criteria for the Review of Multiple Study designs (ICROMs) measure. Studies using quantitative and qualitative methods were included.

In the first stage of the study selection, two reviewers (SB and LC) independently screened the first 100 titles and abstracts using the online review software Rayyan (accessed at https://Rayyan.qcri.org). A Kappa score for the agreement was independently calculated by a third reviewer (SA). Each of the two reviewers then proceeded to screen half of the remaining titles and abstracts.

**Fig. 1:**
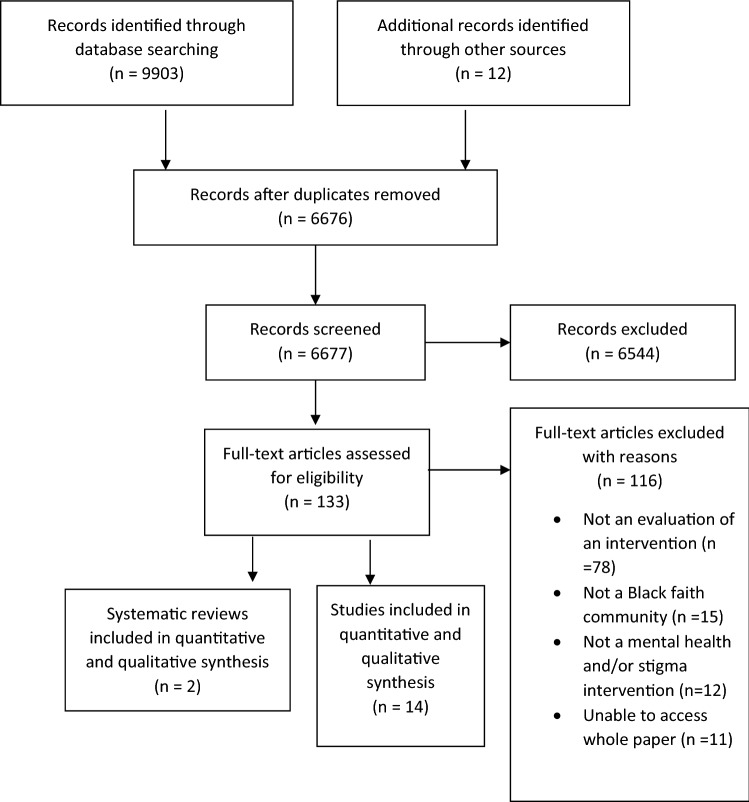
PRISMA Flow Diagram

In the second stage, two reviewers (SB and LC) undertook full-text review of the first 10 selected papers. A third reviewer (SA) helped to resolve any disagreements on inclusion. Following this process, the two reviewers (SB and LC) independently reviewed half of the remaining papers (see Fig. 1 and Table 1.

**Table 1:** Characteristics of included studies

Study	Location	Focused condition	Faith involvement^a^	Target population description^b^	Intervention type	Design	Outcomes	Psychometric properties of outcome measures	Quality assessment	ICROMS risk of bias score^c^
1. Aaron et al. [1]	USA	HIV stigma	Collaborative	Black faith congregation (adults and adolescents), 69	Workshop	Non-controlled before-after	Knowledge score, session attendance, HIV screening rate	Non standardised	Low	12 (22)
2. Anthony et al. [4]	USA	Depression	Faith placed	Black faith leaders, 42	Workshop	Non-controlled before-after	Depression knowledge score (DAQ)	This scale showed good internal consistency: Cronbach’s alpha coefficient was 0.84; satisfactory test–retest reliability: intraclass correlation coefficient was 0.62 (95% C.I. 0.37 to 0.78) [27]	Low	17 (22)
3. Brown et al. [10]	USA	Mental health	Faith placed	Black faith congregation, 110	Workshop	Non-controlled before-after	Mental illness stigma (AQ-SF)	AQ-SF- self report measure of public stigma towards mental illness. A factor analysis yielded alphas that ranged from 0.60 to .93. Intraclass correlations were tested over the course of a week and had test–retest reliability ranging from 0.74 to 0.90 [9] The Level of Familiarity Scale (LOF) assesses how familiar an individual is with mental illness. The psychometric properties of the LOF suggest that it is a reliable and valid measure of familiarity with mental illness [16]	Low	21 (22)
4. Coleman et al. [13]	South Carolina, USA	HIV stigma	Collaborative	Black faith congregation and wider Black community, 30	Prevention programme	Qualitative (1:1 interviews and focus groups)	Thematic	n/a	Adequate	24 (16)
5. Crewe [18]	USA	Mental health	Collaborative/Faith placed	Wider Black community, 228	Workshops	Non-controlled before-after	Knowledge		Low	9 (22)
6. Griffith et al. [25]	Michigan, USA	HIV stigma	Faith based	Black faith leaders and Black faith congregation, 253	Training programme	Non-controlled before-after	Knowledge, stigma	Non-standardised measures	Low	10 (18)
7. Johnson and Van Hecke [32]	Milwaukee, USA	Autism	Faith based	Black faith leaders, 14	Training programme	Non-controlled before-after	Screening and referral, knowledge, attitudes, and self-efficacy		Low	16 (22)
8. Mantovani et al. [34, 35]	London, UK	Mental health	Faith based	Black faith congregation, 13	Training programme	Qualitative (1:1 interviews)	Thematic	n/a	Adequate	25 (16)
9. Mynatt et al. [43]	USA	Depression	Faith based	Black faith congregation, 9	Group support session	Non-controlled before-after	Depression, anxiety, hopelessness, and loneliness scales		Low	15 (22)
10. Suite et al. [52]	New York, USA	Trauma	Faith placed	Black faith congregation, 426	Workshop	Non-controlled before-after	Knowledge		Low	12 (22)
11. Berkley-Patton et al. [6]	Kansas City, USA	HIV stigma	Faith based	Black faith congregation, 543	Multicomponent (outreach, church services, ministry groups)	Cluster randomised controlled trial	Stigma and satisfaction	Religiosity was measured with a summation of the seven-item version of the Religious Background and Behavior Scale HIV Knowledge Questionnaire V stigma items were selected from national studies on HIV stigma [31] – non-standardized	Low	20 (22)
12. Mashamba et al. [37]	Limpopo province, South Africa	HIV stigma	Faith based	Black faith leaders (faith healers), 103	Training programe	Randomised control trial	Knowledge and attitudes	Aids related stigma scale (Kalichman et al. 2005). This scale is internally consistent, alpha = 0.75 and time stable over 3 months, r = 0.67 (Kalichman et al. 2005). This was adapted for use in this study	Adequate	24 (22)
13. Daniels and Archibald [19]	Maryland, USA	Mental health (well-being)	Faith placed	Black faith congregation, 29	Congregation meetings	Qualitative (focus groups)	Thematic	n/a	Low	5 (16)
14. Gum et al. [26]	Florida, USA	Mental health	Collaborative	Black faith congregation, 129	Congregation meeting	Qualitative (focus groups)	Logic model	n/a	Adequate	22 (16)
15. Hankerson and Weissman [28]		Mental health				Systematic review		n/a		
16. Hays and Aranda [29]		Mental health				Systematic review		n/a		

^a^Faith involvement = Faith placed, Faith based, Collaborative

^b^Target population description = Black faith leaders/Black faith congregation/wider Black community, no. of participants

^c^ICROMS risk of bias score suggested minimum for study design

### Data extraction and management

**Fig. 2:**
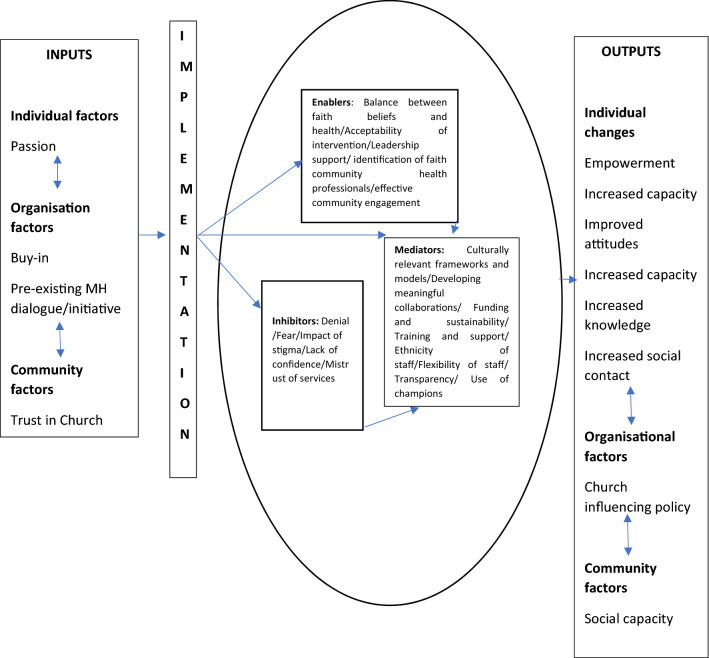
Framework for faith-based mental health awareness and stigma interventions adapted from Coleman et al. 2016.

Review authors (SB and LC) extracted data from all included studies onto standard forms and presented in a “Characteristics of Included Studies table”, the country of origin, health condition, faith involvement, target population (and sample size), intervention type, study design, assessment of quality and ICROMs score (out of the total for study design) [57]. This review has defined faith involvement as either (1) collaborative, (2) faith based, or (3) faith placed. (1) Collaborative refers to co-production between faith group and external organisations, (2) faith based refers to an intervention designed for use in a faith organisation by an external source ef.g., universities and (3) faith placed refers to an intervention initiated by a faith group (Fig. 2 and Table 2)

**Table 2:** Results by category, level and concept

Category	Level and concept
*Inputs* Elements present before the adoption or implementation of the intervention	‘Buy in’ of proposed initiative Pre-existing dialogue around Mental Health or initiatives Trust in the Church
*Enablers* Factors that facilitated the implementation and success of the intervention	Balance between faith beliefs and health Acceptability of intervention Leadership support Identification of faith community health professionals Effective community engagement
*Inhibitors* Factors that functioned as barriers to implementation	Denial of the existence of mental illness Fear of/stigma Impact of stigma Lack of confidence in mental health service Mistrust of mental health services
*Mediators* Factors that influenced the delivery of the intervention	Culturally relevant frameworks and models Development of meaningful collaborations Funding and sustainability of the proposed initiative Training and support of church community members Ethnicity of staff Flexibility of staff in relation to faith communities Transparency of external organisations Use of champions
*Outputs* Changes that were attributed to the intervention	Empowerment Improved attitudes about mental illness Increased capacity around mental illness Increased knowledge about mental illness Increased social contact with people suffering from mental health difficulties Establishment of Social capital Church influencing mental health policy Increased capacity

.

### Qualitative synthesis and analysis

A thematic analysis was undertaken to synthesise the results of the qualitative studies included [53]. A coding frame was developed for the qualitative studies (*n* = 4) using NVivo software by LC and by discussion and data examination (LC, HL and SA). Data were grouped and regrouped into a revised set of inter-related themes and subthemes informed by previous research. Collins et al. [15] created a framework through which community-based organisations could assess capacity and identify needs to implement evidence-based community HIV prevention initiatives. This was explored further by Coleman et al. [13], cited in included studies [14] who modified the framework to demonstrate how community organisations might successfully implement evidence-based intervention specifically in African American faith communities. This framework suggests analytic categories presented broadly as inputs, enablers and inhibitors, mediators, and outputs to guide development. Within this review, a final coding matrix was generated, and a draft synthesizing conceptual model created, based on Coleman et al. [14]. A second author (SA or HL) independently examined the final coding frame for inclusion.

### Quality assessment of included quant/qual studies

Risk of bias was assessed in each study by adapting the Integrated quality Criteria for the Review of Multiple Study designs (ICROMS) tool [57]. Studies were assessed as low quality (does not meet ICROMS criteria for inclusion in a systematic review) or adequate (meets ICROMS criteria for inclusion).

### Risk of bias in included studies

Only four (29%) of the included studies were assessed as having adequate quality according to ICROMs criteria [57]. Most studies included in this review were of low quality and high risk of bias. None of the eight included non-controlled before-after studies met the minimum score. However, three out of the four qualitative studies included in the review were of acceptable quality. Therefore, there is a low risk of bias in the qualitative synthesis.

### Patient and public involvement

Although this research contained no direct and formal public engagement or service user involvement, the impetus for the review was informed by close collaboration with Black faith communities and mental health services by the first author.

## Results

In total 9915 records were identified, 9903 results from database searching and 12 from other sources. After duplicates were removed, there were 6673 original articles for the title and abstract screening. The Kappa score for agreement on inclusion and exclusion of the first 100 titles and abstracts screened was calculated as 0.95. A total of 132 papers were selected for full article review. Overall, 16 papers were included in this review (2 systematic reviews and 14 studies). 116 full-text articles were excluded (see Online Appendix B) and is summarised in the PRISMA diagram (see Fig. 1. No study was excluded based on the quality assessment outcome.

The characteristics of the studies are summarised in Table 1. Of these, 12 studies were from the USA, 10 of which were urban populations. Of the 16 results, 11 (including the two systematic reviews) focused on mental health awareness interventions, including well-being, depression, trauma, and autism. The remaining five studies focused on stigma related to people living with HIV/AIDS. No studies relating to stigma towards people with other health conditions were identified in relation to the Black community. There were different models of faith involvement represented. Of the empirical studies, four were collaborative, six were faith-based and four were faith-placed. Study sample size ranged from nine to 543. The target populations varied amongst empirical studies, with three targeting faith leaders only, eight the Black faith community, and the remainder mixed (for example including the congregation and the wider community).

Within the studies, eight focused on adult working-age populations and most participants were women in eight of the 14 studies. The intervention types included outreach, training, workshops, group support sessions and congregation meetings. Most studies were non-controlled before-after designs (8/14). Of the remainder, four were qualitative (employing individual interviews and focus groups) and two were randomised control trials. Study outcomes were assessed using a range of variables (knowledge, attitudes, stigma, satisfaction) as well as observational data such as attendance and screening rate.

## Results and framework development

### Quantitative results

This review identified ten quantitative studies and two systematic reviews for inclusion. Two studies from the systematic reviews were included within this review. Studies were excluded that did not meet criteria for this review, e.g., focused on multiple ethnic groups. The systematic reviews explored church-based health programmes for mental disorders aiming to reduce symptoms [28] and a faith-based mental health intervention aiming to improve symptoms [29]. Distinct from this review, the target group of the reviews was African Americans, and a reduction of mental health symptoms was a pre-requisite for inclusion. Hays and Aranda [29] prioritised solely quantitative studies with post-intervention results, using standardised clinical assessment measures or self-report ratings. Whilst Hankerson and Weissman [28] included qualitative reviews, studies in which all participants were pastors/clergy and descriptive studies (studies that did not include data) were excluded. The authors acknowledged that this may have limited findings. Significantly, Hankerson and Weissman [28] observed the importance of church-based health promotion programs (CBHPP) as an effective way of reducing health disparities amongst African Americans.

Hays and Aranda [29] similarly noted that faith-based interventions offer a culturally sensitive and accessible way to address mental health difficulties in the African American population. Both studies demonstrated that interventions within faith communities can help to reduce current mental health symptoms [28], 29 and offer a more culturally accessible way of addressing mental health in this population.

### Effectiveness of Interventions

Included studies adopted didactic training or workshop approaches to deliver mental health awareness and/or stigma reduction information. There was a consistent absence of detail around models of illness used across studies. The outcome measures of included studies predominantly measured change in knowledge (7/10). Three studies included measures of stigma [6], Brown et al. [25]; and two included evaluation of attitudes [32, 37]. One study was conducted outside of the USA [37] and the result of a collaboration between faith and external organisations [1, 14, 18]. Half of the included studies focused directly on mental health issues, whilst the remaining studies explored HIV stigma reduction initiatives. In four studies, faith leaders were specifically targeted,six studies involved members of the congregation and all interventions were group based.

A total of four studies measured stigma directly and one (Brown et al. [10]) used a standardised questionnaire. The Attribution Questionnaire [9] is a self-report measure of public stigma towards mental illness. A factor analysis yielded alphas that ranged from 0.60 to 0.93 and test–retest reliability ranging from 0.74 to 0.90 [9]. The other stigma outcome measures used included non-standardised stigma questionnaires [6], Griffiths et al. 2010) and adaptations of previously standardised questionnaires [37].

Of the ten quantitative studies, eight were non-controlled, pre-, and post-design and two were randomised controlled studies. Low ratings of quality were given to nine studies; one was rated as adequate. Mashamba et al. [37] developed a 2-day training manual and evaluated the knowledge, attitudes, and management of faith healers of Apostolic churches before and after they attended an HIV and AIDS training programme. The training was designed around the Information-Motivation -Behaviour skills model [21], which suggests that behaviour change occurs when individuals’ have enough information, personal and social motivation, and behavioural skills. Mashamba et al. [37] included a randomised controlled trial design with faith healers systematically assigned to the control or intervention group and a two-month follow-up.

Outcome measures were adapted from those used in previous studies, but whether these are standardised remains unclear. The study reported overall improvements in knowledge but not behaviour domains [37]. The authors noted limitations, which included one post-training follow-up impact assessment and the need for training to be longer than two days to effect sustainable change. They concluded that training for faith communities needs to be systematic and evaluated as part of a randomised controlled trial. It was noted that faith groups can offer a positive contribution to community-level education and prevention initiatives [37].

### Qualitative results

Three [14, 26, 34, 35] of the four qualitative studies included were rated as ‘adequate’ [57]. Daniels and Archibald [19] were rated as ‘low’ [57]. The studies rated as ‘adequate’ included a robust qualitative methodology whilst the study rated ‘low’ was predominantly descriptive. Within this arm of the review, the thematic analysis of the four identified qualitative studies was conducted. Five categories were identified through thematic analysis.

### Effective ingredients of interventions

A number of ‘effective ingredients’ utilised in mental health awareness or stigma reduction interventions have been identified in this review. Within the enabler theme, a sub-theme ‘acceptability of the intervention’ was identified. This requires the community organisations involved to ensure that interventions are acceptable and accessible to the local population, “*Bottom-up approaches to engagement are supposed to provide culturally appropriate ways to communicate health messages*” [34, 35], p. 173) and community engagement, “*Community engagement approaches have become a growing component in the British public health system with community health champions becoming a favoured method of engagement to address a variety of issues*.” [34, 35], p. 168).

Culturally relevant frameworks are a sub-theme identified within the ‘mediator’ theme, which acknowledges the creation of a model to capture the *“reciprocal relationship between community engagement processes and the social determinants of mental health”* [34, 35], p. 169).

An additional sub-theme associated with the effective ingredients of interventions is the vital role of ‘champions’, that will ensure the successful delivery of interventions, “*Lay workers have a unique advantage in accessing hard-to-reach groups as they are embedded in local community networks*” (Tran et al. 2014 cited in [34, 35], p. 168).

### Factors influencing implementation

Several factors were found to influence the implementation of mental health awareness and/or stigma reduction interventions with Black faith communities. In the ‘input’ theme, the sub-theme ‘buy-in’ refers to the importance of having senior members of the Church invested in the intervention; studies indicated that without this the potential success of implementation would be undermined; *“…. both religious leaders and congregation members needed to buy-in and contribute to the program for it to be successful.”* [13], p. 122) The sub-theme pre-existing dialogue indicates that implementation is improved when faith communities have already begun conversations about mental health and are open to mental health as a concept,*“Having a pre-existing infrastructure, such as a health ministry, into which a prevention program could fit was important”* [13], p. 122). In Gum, et al. [26] the success of a senior wellness programme model was in part being able to “‘*build upon churches’ strengths, including existing spiritual, health and senior ministries”* [26], p. 227). The final sub-theme is trust in the Church and refers to the trust and confidence that the faith community has in the Church and Church leaders, transferred from members of the congregation to the intervention, “*The church has always been a place we can go for information, especially when it came to things like health. It was trusted there*.” [13], p. 120).

In the ‘enabler’ theme, a balance between faith beliefs and health describes how the faith community incorporate mental health within their faith doctrine, *“A lot of churches struggle between where you draw the line between compassion and endorsing a particular sort of behaviour. That’s where Christians as a whole struggle and some churches are still trying to find that balance.”* [13], p. 121) and “*adoption and implementation of future HIV/AIDS prevention programs must be undertaken with care and respect for the church as an institution”* [13], p. 122). The final sub theme is the identification of faith community health professionals and implies to find members in the faith community who are also health professionals and harness their expertise to help drive implementation, “One care team member noted*, ‘‘it’s because I was able to talk about it [HIV/AIDS] more freely,’’ that she was able to take the lead.”*; *“*a pastor observed*, ‘‘I realize there are members of my congregation that have gifts and talents that I cannot touch. So, in the areas where I need them, I utilize them.’’* [13], p. 119).

Within the ‘inhibitor’ theme, five sub-themes emerged. The first sub-theme is denial, *“They did ‘not want to talk about it’, thereby creating ‘a block’ to engagement: At the beginning people are difficult. As they hear ‘mental health’ for them it is madness, and they don’t want to know.”* [34, 35], p. 171) and is associated with further sub-themes of fear and the impact of stigma. *“Fear and stigma and a kind of self-loathing, I think. They are the three things that have bedevilled the black community.”* [34, 35], p. 172),*“Fear was a salient part of stigma in this study.”* [13], p. 122). A further sub theme was identified as lack of confidence and outlines how faith community members are unskilled to take part in prevention initiatives*, “participants felt they did not have the tools to be able to confidently broach the subject of mental health”* [34, 35], p. 172). The final sub-theme relates to a sense of mistrust in the treatment that service users are likely to receive from mental health services, *“African-American men tend to be very distrustful of the United States service delivery and health care system because of historical evidence of racial discrimination”* [19], p. 2).

Sub-theme factors within the ‘mediator’ theme found to impact delivery of intervention included developing meaningful collaborations, *“both Church representatives and collaborating community service providers should be involved in building the infrastructure for the program and delivering program activities.”* [26], p. 227) and being transparent within these relationships.

One dominant sub-theme was about funding and sustainability. Coleman et al. [13], p.12) noted that*, “The contexts of policy and stigma that emerged in this study reinforce the need to support African American churches in the provision of long-term sustainable solutions in their communities”* and commented that*, “Participants feared that an abrupt end to funding would derail their efforts for a long time.”*. Another sub-theme that is linked to funding and sustainability is the need for training and support to help embed interventions. Mantovani et al. [34, 35], p. 173) found that *“training and support are critical to translating these approaches (engagement programmes) into practice”.*

Ethnicity and flexibility of staff working on the intervention are additional sub-themes. Black faith communities *“favour mental health resource centres staffed with individuals from the same ethnic groups who look ‘at the world from their clients’ perspective’* and *‘share something of themselves’* [34, 35], p. 168). It is vital that staff involved in delivering interventions to Black faith communities can be flexible*, “Mental health professionals should feel encouraged to develop ties with their local Church communities, as long as they take a holistic culturally competent approach. The range of activities may stretch mental health providers’ traditional terrain, so it is important to remain flexible about the scope of one’s practice…”* [26], p. 230*)*.

Within the ‘output’ theme, seven sub-themes emerged, firstly empowerment. Community members who participated in the delivery of an intervention to their faith community *“spoke of a sense of empowerment resulting from their participation. Not only could they now take control over their own health and mental health but also with the knowledge and techniques acquired, they could help others”* [34, 35], p. 172). There was also reported increase in knowledge*, “11 participants had acquired more knowledge of the conditions that contributed to maintaining and improving mental well-being”* [34, 35], p. 172),attitudes*, “Let’s tell the truth for what it is so we can educate the next generation that’s coming up.’’* [13], p. 121) and willingness to have social contact, *“… let them see it’s not a matter of how you were infected, it’s that you are infected. Let them see the face of AIDS.”* [13], p. 121).

Three sub-themes emerged around increased capacity, the role the Church can play to influence policy and social capital. These themes capture the significance of the Church as an organisation to help provide *a “bridge with public services; five reported referral practices to primary care services (three to IAPT* (Improving Access to Psychological Therapy) *services and two to general practitioners)”* and have a key role in recent UK government initiatives *to “invest in local communities’ involvement in health-related activities for those experiencing disadvantage…. a promising way to reduce the social gradient”* [36] cited in [34, 35], p. 173).

## Discussion

Evidence for the effectiveness of interventions was observed within the quantitative findings however these were predominantly rated as low quality according to the ICROMS criteria [57]. These studies noted the effectiveness of a manualised training intervention [37] and the need for effective interventions to include standardised measures particularly of stigma, post-training impact assessments, the need for more high-quality randomised control trials focused on Black faith communities and the importance of using rigorous qualitative methods to ensure the quality of intervention studies.

The active ingredients of mental health and/or stigma reduction interventions to promote mental health and reduce stigma in Black faith communities included the importance of developing bottom-up approaches to engagement, which harness community expertise to develop acceptable and accessible interventions [34, 35]. The importance of including champions to engage communities was also acknowledged [34, 35]. Finally, factors found to influence the effective implementation of mental health awareness and/or stigma reduction intervention for Black faith communities included buy in from faith leaders [13], utilisation of existing mental health networks within faith communities [26] and trust in the Church [13].

Factors influencing the implementation of interventions were related to attitudes of Church attendees and included fear, denial, stigma, and mistrust in mental health services [19, 34, 35]. The remaining factors identified were associated with the delivery of the intervention and included the development of a meaningful collaboration between external and faith organisations, secure funding to sustain the intervention beyond the remit of the study and the ethnicity and flexibility of staff [13, 26, 34,35].

Within this review, stigma has been defined as a problem of (i) knowledge (ignorance); (ii) attitudes (prejudice); and (iii) behaviours (discrimination) [54]. Berkley-Patton et al. [6] discussed ‘stigma beliefs’ and all other studies which directly referenced stigma [1, 13, 25, 37] mentioned decreasing stigma but none included a definition of stigma. It is interesting that none of the outcome measures included assessment of changes across all three of the stigma dimensions identified by Thornicroft et al. [54]. Fufrthermore, it is noticeable that the focused stigma reduction interventions targeted the stigma of HIV infection rather than mental illness.

Only one study that focused on mental health included a stigma outcome measure (Brown et al. [10]). The observed emphasis on stigma reduction in physical health conditions in Black faith communities could suggest that addressing physical health seems more acceptable within this community, whereas mental illness remains a taboo. Limited conclusions about the efficacy of the interventions can be drawn from the results of the quantitative studies, due to the high risk of bias. However, the inclusion of these studies in this review demonstrates the scope of the existing literature, different types of faith community involvement, range of conditions, target populations and types of intervention.

The quantitative studies included in this review found that the literature in this field largely originates from the USA. One was from South Africa, one from the UK and two from the USA. The authors found that the evidence for interventions in Black faith communities and the ‘active ingredients’ of such interventions are influenced by strong inter-relationships between inputs, enablers, inhibitors, mediators and outputs. To illustrate these relationships, this review tentatively adapted the conceptual model based on Coleman et al. [14] and highlighted the ‘active ingredients’ for each of these five model components. Inclusion of models to shape and guide the development of studies was present in all four of the ‘adequate’ studies [13, 26, 34,35, 37]. This preliminary model may be useful for understanding this area based on the available research however, further research is required to progress its development.

The use of models can potentially be added to the ‘active ingredients’ for a successful intervention with the Black faith community. Models can help for example to explain changes, behaviours and a theoretical foundation enables the researcher to select appropriate outcome measures [50]. Sales et al. [50] identified the use of models as a critical step in the development of interventions and the implementation of evidence-based care.

Two included studies were systematic reviews [28, 29]. Half of the studies included in Hankerson and Weissman [28] were RCTs whereas Hays and Aranda [29] documented only one RCT. Hays and Aranda [29] included interventions which sought to treat or reduce symptoms, provide skills to prevent symptoms and increased knowledge of the mental illness. Similarly, Hankerson and Weissman [28] included studies that were concerned with education and prevention of mental health diagnoses,these studies examined the delivery of culturally tailored adaptations of established interventions (e.g., smoking cessation, substance misuse reduction) within Churches. Hankerson and Weissman [28] included studies that were concerned with education and prevention of mental health diagnoses,these studies examined the delivery of culturally tailored adaptations of established interventions (e.g., smoking cessation, substance misuse reduction) within Churches. In contrast, Hays and Aranda [29] focused on studies exploring the scope, effectiveness, and sociocultural relevance of faith-based interventions for African American communities. One study identified by the search strategy for this review was excluded as it could not be evaluated using the ICROMs. It is possible to infer from these findings that the current evidence for mental health/and or stigma reduction interventions in Black faith communities is more likely to include robust research methodology in instances where the faith setting is utilised to facilitate the delivery of an intervention as opposed to studies which work collaboratively with faith communities.

The importance of establishing partnerships and collaboration is emphasised by Hays and Aranda [29] and within the findings of this review. Development of partnership working has been found to increase buy-in, motivation and commitment to the initiative [13], which may increase the likelihood of sustainability and the opportunity to create trusting relationships between mental health services and local Black faith communities. Gum et al. [26], p. 222) commented on the “*valuable contribution of a community based collaborative approach, as opposed to a more traditional approach driven by researchers’ “expertise.*” It is vital that mental health professionals and researchers have an awareness of the key principles of effective community engagement. The National Institute for Health and Care Excellence (NICE, 2016), guidelines for community engagement outline best practice recommendations. The findings from this review support and highlight the importance of developing partnerships and collaborations with the local community, building on existing community strengths and resources, co-production and community-based participatory research.

## Strengths and limitations

This review was strengthened by a thorough formal systematic review search strategy, the utilisation of many databases and hand-searching of additional relevant references. The authors used multiple reviewers and validation strategies to be confident in the synthesised data.

One strength is the tentative adaptation of a conceptual model to fit with the Black faith community. It is possible that this can be tested against future research in this area. We acknowledge that this framework can only be viewed as a preliminary attempt to understanding this area and would need to be substantiated by further studies.

To reduce risk of bias, the authors focused on peer-reviewed papers. It is possible that by not including the grey literature the team may have lost valuable data. Grey literature was not searched due to limited resources and time. Furthermore, several papers had to be excluded from the systematic review as the authors were unable to reach the authors by direct email or telephone.

The decision to search for health stigma interventions other than just those for mental health stigma in Black faith communities was based on the expectations that: (1) the literature on mental health stigma interventions in these settings would be sparse; and (2) we would thus identify active ingredients and implementation factors for interventions within Black faith community setting which are common across health stigma related interventions more broadly. For example, the importance of faith community leadership support, ensuring that the intervention is driven by culturally relevant frameworks and models and the importance of having the same ethnicity for staff as for participants.

A notable limitation to this study is the limited range of countries in which included studies were set. Given that this review was interested in UK populations, it is a significant limitation that only 1 of the 16 studies was based within the UK. The majority of the studies (12/14) were from the USA which may limit the generalisability of findings.

There was an absence across all studies of strategies to improve mental health awareness and/or reduce the stigma of alternatives to a training or workshop delivery format and details of which model of illness was presented to participants. Further, no studies reported using contact and reducing social distance.

## Recommendations

Conducting formal RCTs in community settings where clinical trials may be unfamiliar and potentially unacceptable presents a challenge for researchers. This review considers whether a more accessible approach may be a cluster RCT which would involve the randomisation of whole churches/congregations rather than individuals. The presented conceptual model offers an opportunity to present the ‘active ingredients’ to be considered for researchers who want to embark upon the future development of interventions for Black faith communities.

Further, this review emphasises the importance of applying effective community engagement strategies to develop close partnerships and collaborations with the local community to address healthcare inequalities for minority populations.

This review aimed to examine the effectiveness of interventions; however, our ability to achieve this aim was limited as only one of the quantitative studies was ‘adequate’. The lack of UK-based research about specific mental health awareness and/or stigma reduction interventions for Black faith communities in comparison to the USA suggests an urgent need for further robust, evidence-based studies to be conducted within this population.

## Conclusions

The results of this review suggest that mental health awareness and/or stigma reduction interventions in Black faith communities initially requires buy-in from faith communities, motivation and where possible engagement with faith groups who have already begun their own mental health initiatives. The intervention will benefit the communities if there are members of health professions within congregations who can help disseminate relevant information and encourage engagement. Co-production and partnership working are key to ensure that an acceptable and accessible intervention is created. External organisations embarking on this work need to be proficient in community engagement skills. It is imperative that there is an awareness of the impact of mistrust of mental health services, including stigma and discrimination around mental illness within Black African and Caribbean communities, which may create a barrier to effective implementation of an intervention. To mitigate such an obstacle, researchers may benefit to employ culturally relevant frameworks, work flexibly and with transparency and promote champions to facilitate engagement. Whilst this paper cannot demonstrate currently a strong evidence base for effective work in this area and has identified the need for robust research studies in this area, the systematic review can shed light on the lessons learnt and necessary key requirements for interventions that can guide future projects.

## Supplementary Information

Below is the link to the electronic supplementary material.

Supplementary file1 (DOCX 24 kb)

## References

[1] Aaron E, Yates L, Criniti S (2011). A collaborative HIV prevention and education initiative in a faith-based setting. J Assoc Nurses AIDS Care.

[2] Anderson KK, Fuhrer R, Schmitz N, Malla AK (2013). Determinants of negative pathways to care and their impact on service disengagement in first-episode psychosis. Social Psychiatry and Epidemiology.

[3] Ajnakina O, Lally J, Di Forti M, Kolliakou A, Gardner-Sood P, Lopez-Morinigo J, Vassos E (2017). Patterns of illness and care over the 5 years following onset of psychosis in different ethnic groups: the GAP-5 study. Soc Psychiatry Psychiatr Epidemiol.

[4] Anthony JS, Morris E, Collins CW, Watson A, Williams JE, Ferguson B, Ruhlman DL (2016). Equipping African American Clergy to recognize depression. J Christ Nurs.

[5] Barnett P, Mackay E, Matthews H, Gate R, Greenwood H, Ariyo K, Bhui K, Halvorsrud K, Pilling S, Smith S (2019). Ethnic variations in compulsory detention under the Mental Health Act: a systematic review and meta-analysis of international data. Lancet Psychiatry.

[6] Berkley-Patton JY, Moore E, Berman M, Simon SD, Thompson CB, Schleicher T, Hawes SM (2013). Assessment of HIV-related stigma in a US faith-based HIV education and testing intervention. J Int AIDS Soc.

[7] Bhui K, Stansfeld S, Hull S, Priebe S, Mole F, Feder G (2003). Ethnic variations in pathways to and use of specialist mental health services in the UK. Br J Psychiatry.

[8] Blank MB, Mahmood M, Fox JC, Guterbock T (2002). Alternative mental health services: the role of the Black church in the South. Am J Public Health.

[9] Brown SA (2008). Factors and measurement of mental illness stigma: a psychometric examination of the Attribution Questionnaire. Psychiatr Rehabil J.

[10] Brown JF (2009). Faith-based mental health education: a service-learning opportunity for nursing students. J Psychiatr Ment Health Nurs.

[11] Codjoe L, Byrne M, Lister M, McGuire P, Valmaggia L (2013). Exploring perceptions of “wellness” in Black ethnic minority individuals at risk of developing psychosis. Behav Cogn Psychother.

[12] Codjoe L, Barber S, Thornicroft G (2019). Tackling inequalities: a partnership between mental health services and black faith communities. J Ment Health.

[13] Coleman JD, Lindley LL, Annang L, Saunders RP, Gaddist B (2012). Development of a framework for HIV/AIDS prevention programs in African American churches. AIDS Patient Care STDS.

[14] Coleman JD, Tate AD, Gaddist B, White J (2016). Social determinants of HIV-related stigma in faith-based organizations. Am J Public Health.

[15] Collins CE, Whiters DL, Braithwaite R (2007). The Saved SISTA Project: A faith-based HIV prevention program for Black women in addiction recovery. Am J Health Stud.

[16] Corrigan PW, Markowitz FE, Watson A, Rowan D, Kubiak MA (2003). An attribution model of public discrimination towards persons with mental illness. J Health Soc Behav.

[17] Clement S, Schauman O, Graham T, Magionni F, Evans-Lacko S, Bezborodovs N, Morgan C, Brown JSL, Thornicroft G (2015). What is the impact of mental health-related stigma on help-seeking? A systematic review of quantitative and qualitative studies. Psychol Med.

[18] Crewe SE (2007). Joy of living: a community-based mental health promotion program for African American elders. J Gerontol Soc Work.

[19] Daniels K, Archibald P (2011). Merging community and faith-based organizations to empower African American males. J Pastoral Care Counsell.

[20] Egede LE, Walker RJ (2020). Structural racism, social risk factors, and Covid-19—a dangerous convergence for Black Americans. N Engl J Med.

[21] Fisher WA (1997). A theory-based framework for intervention and evaluation in STD/HIV prevention. Canad J Hum Sexual.

[22] Ghali S, Fisher HL, Joyce J, Major B, Hobbs L, Soni S, Johnson S (2013). Ethnic variations in pathways into early intervention services for psychosis. Br J Psychiatry.

[23] Gajwani R, Parsons H, Birchwood M, Singh SP (2016). Ethnicity and detention: are Black and minority ethnic (BME) groups disproportionately detained under the Mental Health Act 2007?. Soc Psychiatry Psychiatr Epidemiol.

[24] Gopalkrishnan N (2018). Cultural diversity and mental health: considerations for policy and practice. Front Public Health.

[25] Griffith DM, Pichon LC, Campbell B, Allen JO (2010). Your blessed health: a faith based CBPR approach to addressing HIV/AIDS among African Americans. AIDS Educ Prev.

[26] Gum AM, Watson MA, Smith BA, Briscoe R, Goldsmith J, Henley B (2012). Collaborative design of a church-based, multidimensional senior wellness program by older adults, church leaders, and researchers. J Religion Spiritual Aging.

[27] Haddad M, Menchetti M, McKeown E, Tylee A, Mann A (2015). The development and psychometric properties of a measure of clinicians’ attitudes to depression: the revised Depression Attitude Questionnaire (R-DAQ). BMC Psychiatry.

[28] Hankerson SH, Weissman MM (2012). Church-based health programs for mental disorders among African Americans: a review. Psychiatr Serv.

[29] Hays K, Aranda MP (2016). Faith-based mental health interventions with African Americans: a review. Res Social Work Pract.

[30] Henderson C, Robinson E, Evans-Lacko S, Corker E, Rebollo-Mesa I, Rose D, Thornicroft G (2016). Public knowledge, attitudes, social distance and reported contact regarding people with mental illness 2009–2015. Acta Psychiatr Scand.

[31] Herek GM, Capitanio JP, Widaman KF (2002). HIV-related stigma and knowledge in the United States: prevalence and trends, 1991–1999. Am J Public Health.

[32] Johnson N, Van Hecke A (2015). Increasing Autism awareness in inner-city churches: a brief report. J Pediatr Nurs.

[33] Logie CH (2020). Lessons learned from HIV can inform our approach to COVID-19 stigma. J Int AIDS Soc.

[34] Mantovani N, Pizzolati M, Gillard S (2017). Engaging communities to improve mental health in African and African Caribbean groups: a qualitative study evaluating the role of community well-being champions. Health Soc Care Community.

[35] Mantovani N, Pizzolati M, Edge D (2017). Exploring the relationship between stigma and help-seeking for mental illness in African-descended faith communities in the UK. Health Expect.

[36] Marmot M, Allen J, Goldblatt P, Boyce T, McNeish D, Grady M, Geddes I (2010). Fair Society, Healthy Lives (The Marmot Review). Institute of Health Equity. http://www.instituteofhealthequity.org/resources-reports/fair-society-healthy-lives-the-marmot-review

[37] Mashamba T, Peltzer K, Maluleke TX, Sodi T (2011). A controlled study of an HIV/AIDS/STI/TB intervention with faith healers in Vhembe District, South Africa. Afr J Tradit Complement Altern Med.

[38] Memon A, Taylor K, Mohebati LM, Sundin J, Cooper M, Scanlon T, de Visser R (2016). Perceived barriers to accessing mental health services among black and minority ethnic (BME) communities: a qualitative study in Southeast England. BMJ Open.

[39] Moher D, Liberati A, Tetzlaff J, Altman DG (2009). Preferred reporting items for systematic reviews and meta-analyses: the PRISMA Statement. BMJ.

[40] Morgan C, Dazzan P, Morgan K, Jones P, Harrison G, Leff J, AESOP study group (2006). First episode psychosis and ethnicity: Initial findings from the AESOP study. World Psychiatry.

[41] Morgan C, Webb RT, Carr MJ, Kontopantelis E, Green J, Chew-Graham CA, Ashcroft DM (2017). Incidence, clinical management, and mortality risk following self harm among children and adolescents: Cohort study in primary care. BMJ (Online).

[42] Mulholland H (2017). Jacqui Dyer: talking about race and mental health is everyone’s business. The Guardian. Retrieved from https://www.theguardian.com [Google Scholar]

[43] Mynatt S, Wicks M, Bolden L (2008). Pilot Study of INSIGHT Therapy in African American Women. Arch Psychiatr Nurs.

[44] National Institute for Care Excellence (2016) Community engagement: Improving health and wellbeing and reducing health inequalities (NICE Guideline No. NG440). Retrieved from https://www.nice.org.uk/guidance/ng44

[45] National Institute for Mental Health in England (2003). Inside outside: improving mental health services for black and minority ethnic communities in England.

[46] Ouzzani M, Hammady H, Fedorowicz Z, Elmagarmid A (2016). Rayyan App (Version 0.1.0) [Web application software]. Retrieved from https://Rayyan.qcri.org

[47] Qassem T, Bebbington P, Spiers N, McManus S, Jenkins R, Dein S (2015). Prevalence of psychosis in black ethnic minorities in Britain: analysis based on three national surveys. Soc Psychiatry Psychiatr Epidemiol.

[48] Rogers A (2013) Being built together: a story of new Black Majority Churches in the London Borough of Southwark, London. University of Roehampton. https://www.roehampton.ac.uk/globalassets/documents/humanities/being20built20togethersb203-7-13.pdf

[49] Sainsbury Centre for Mental Health (2002) Breaking the circles of fear: a review of the relationship between mental health services and African and Caribbean communities. https://www.centreformentalhealth.org.uk/sites/default/files/breaking_the_circles_of_fear.pdf

[50] Sales A, Smith J, Curran G, Kochevar L (2006). Models, strategies, and tools: theory in implementing evidence-based findings into health care practice. J Gen Intern Med.

[51] Sohler NL, Bromet EJ, Lavelle J, Craig TJ, Mojtabai R (2004). Are there racial differences in the way patients with psychotic disorders are treated at their first hospitalization?. Psychol Med.

[52] Suite DH, Rollin SA, Bowman JC, La Bril RD (2007). From fear to faith: efficacy of trauma assessment training for New York-based Southern Baptist Church group. Res Social Work Pract.

[53] Thomas J, Harden A (2008). Methods for the thematic synthesis of qualitative research in systematic reviews. BMC Med Res Methodol.

[54] Thornicroft G, Rose D, Kassam A, Sartorius N (2007). Stigma: ignorance, prejudice, or discrimination. Br J Psychiatry.

[55] Time to Change (2010). Family matters: a report into attitudes towards mental health problems in the South Asian community in Harrow, North West London*.* https://www.time-to-change.org.uk.

[56] Wright A, McGorry PD, Harris MG, Jorm AF, Pennell K (2006). Development and evaluation of a youth mental health community awareness campaign—the compass strategy. BMC Public Health.

[57] Zingg W, Castro-Sanchez E, Secci FV, Edwards R, Drumright LN, Sevdalis N, Holmes AH (2016). Innovative tools for quality assessment: integrated quality criteria for review of multiple study designs (ICROMS). Public Health.

