# Supplementary material for: Evidence for interventions to promote mental health and reduce stigma in Black faith communities: systematic review
Source: Soc Psychiatry Psychiatr Epidemiol. 2021 Apr 18;56(6):895–911. doi: 10.1007/s00127-021-02068-y (PMC8053235; doi:10.1007/s00127-021-02068-y)
Supplement: Supplementary file 1 — Supplementary file1 (DOCX 24 kb) [file 127_2021_2068_MOESM1_ESM.docx]

Web Appendix 1.

|  | TEXT WORDS (included in the same way in each database) | SUBJECT HEADINGS (explored in every database, included where available, adapted as appropriate) | Databases | | | | | | | | | | |
| --- | --- | --- | --- | --- | --- | --- | --- | --- | --- | --- | --- | --- | --- |
|  |  |  | ASSIA | BNI | IBSS | SPP | Medline | Psych INFO | Cochrane library | CINAHL | SciLEO | SSCI | Global Health |
| A | Black community related terms |  |  |  |  |  |  |  |  |  |  |  |  |
|  | Black* | Black communities | - | - | - | - | - | - | - | - | - | - | - |
|  | BME | African continental ancestry group / people / communities | x | x | x | - | x | x | x | - | - | - | - |
|  | Africa* | African American / Canadian / Caribbean | x | x | x | - | x | - | x | - | - | - | x |
|  | Caribbean* | Black culture | x | x | x | - | - | - | - | - | - | - | - |
|  |  | Black perspectives | x | - | - | - | - | - | - | - | - | - | - |
|  |  | Black / black people | x | - | x | - | - | x | - | x | - | - | x |
| B | Faith community related terms |  |  |  |  |  |  |  |  |  |  |  |  |
|  | Relig* | Religion / religious belief / religious orthodoxy | x | x | x | - | x | x | x | x | - | - | x |
|  | Spiritual* | Faith | x | x | x | - | - | x | - | - | - | - | - |
|  | Church* | Spirituality | x | x | x | - | x | x | x | x | - | - | - |
|  | Clergy* | Religious organisation / group / community | x | x | x | - | - | x | - | - | - | - | - |
|  | Mosque | Faith based organisation / group / community | - | - | x | - | x | x | x | - | - | - | - |
|  | Faith* | Black church | x | x | x | - | - | - | - | - | - | - | - |
|  | Pastor | Church / churches | x | - | - | - | - | - | - | x | - | - | x |
|  | Christian* | Christianity | x | x | x | - | x | x | x | x | - | - | - |
| C | Mental health related terms |  |  |  |  |  |  |  |  |  |  |  |  |
|  | mental health* or mental illness or mental condition* or mental disabilit* or mental disorder* or mental disease* or mental impair* or mental problem* or mental stress or mental wellbeing or well being | Mental disorders / mental illness | x | x | x | - | x | x | x | x | - | - | x |
|  | psych* health* or psych* illness or psych* condition* or psych* disabilit* or psych* disorder* or psych* disease* or psych* impair* or psych* problem* or psych* stress or psych* wellbeing | Mental health | x | x | x | - | x | x | x | x | - | - | x |
|  |  | Mental Health Services / organisations | x | - | - | - | x | x | x | x | - | - | - |
|  |  | Mental health care | x | x | x | - | - | - | - | x | - | - | - |
|  |  | Mental health professionals / nursing | x | x | x | - | - | x | x | x | - | - | - |
|  |  | Mental health promotion | x | - | - | - | - | - | - | x | - | - | - |
|  |  | Mental health treatment | - | - | - | - | - | - | - | x | - | - | - |
| D | Stigma related terms |  |  |  |  |  |  |  |  |  |  |  |  |
|  | stigma* | Stereotyping | x | x | x | - | x | - | x | x | - | - | - |
|  | discriminat* | Stigma / stigmatization | x | x | x | - | x | - | x | x | - | - | x |
|  | label* | Prejudice | x | x | x | - | x | - | x | x | - | - | - |
|  | stereotyp* | Attitude | x | x | x | - | x | - | x | x | - | - | x |
|  | social distance | Discrimination | x | x | x | - | - | - | x | x | - | - | x |
|  | marginili* | Stigma / stigmatization | x | x | x | - | - | x | - | - | - | - | x |
|  | stigma resilience | Social approval | x | - | - | - | - | x | - | - | - | - | - |
|  | stigma change |  |  |  |  |  |  |  |  |  |  |  |  |
|  | attitud* |  |  |  |  |  |  |  |  |  |  |  |  |
| E | Intervention related terms |  |  |  |  |  |  |  |  |  |  |  |  |
|  | initiative* | Intervention | x | x | x | - | - | x | - | x | - | - | x |
|  | scheme* | Clinical trial | x | x | x | - | x | x | x | x | - | - | x |
|  | project* | Study | - | - | - | - | - | - | - | - | - | - | - |
|  | intervention* | Evaluation | x | x | x | - | x | x | x | x | - | - | x |
|  | evaluat* | Follow up studies | x | - | - | - | x | x | x | x | - | - | - |
|  | prevent* | Randomised controlled trial | x | - | - | - | x | - | - | x | - | - | x |
|  | service* | Comparative study | x | x | x | - | x | - | x | x | - | - | - |
|  | cohort* | Prevention and control | - | - | - | - | - | - | - | - | - | - | - |
|  | Before and after |  |  |  |  |  |  |  |  |  |  |  |  |
|  | Pre-and post |  |  |  |  |  |  |  |  |  |  |  |  |
|  | course* |  |  |  |  |  |  |  |  |  |  |  |  |
|  | program* |  |  |  |  |  |  |  |  |  |  |  |  |
|  | curriculum |  |  |  |  |  |  |  |  |  |  |  |  |

Where x = subject heading or equivalent included in search

Web Appendix 2. Description of features of included articles

| **Feature** | **Sample No.** | **%** |
| --- | --- | --- |
| **Population description** |  |  |
| Black faith leaders | 2 | 14.3 |
| Black congregation | 3 | 21.4 |
| Wider Black faith community | 9 | 64.3 |
| **Population gender (majority)** |  |  |
| Male | 2 | 14.3 |
| Female | 8 | 57.1 |
| Not specified | 4 | 28.6 |
| **Location** |  |  |
| USA | 12 | 85.8 |
| Africa | 1 | 7.1 |
| UK | 1 | 7.1 |
| **Target conditions** |  |  |
| HIV/AIDS | 5 | 35.8 |
| Depression | 1 | 7.1 |
| Mental health (general) | 8 | 57.1 |
| **Population setting** |  |  |
| Rural | 1 | 7.1 |
| Urban | 12 | 85.8 |
| Not specified | 1 | 7.1 |
| **Outcome measures** |  |  |
| Yes | 10 | 71.4 |
| No | 4 | 28.6 |
| **Outcome measured and improved** |  |  |
| Stigma reduction | 2 | 14.2 |
| Knowledge | 8 | 57.1 |
| Attitudes | 1 | 7.1 |
| Engagement | 4 | 28.6 |
| **Target Age Group** |  |  |
| Child and Adolescent | 0 | 0 |
| Adult | 8 | 57.1 |
| Elderly?>65 years of age | 2 | 14.3 |
| Mixed age group | 3 | 21.4 |
| Not specified | 1 | 7.1 |
| **Number of Participants** |  |  |
| 0-50 | 6 | 42.9 |
| 51-100 | 3 | 21.4 |
| 101-150 | 1 | 7.1 |
| 151-200 | 0 | 0 |
| 200+ | 4 | 28.6 |
| **Faith Involvement** |  |  |
| Faith- placed | 4 | 28.6 |
| Faith- based | 6 | 42.9 |
| Collaborative | 4 | 28.6 |
| **Program scope** |  |  |
| Congregation | 3 | 21.4 |
| Community | 4 | 28.6 |
| Region | 5 | 35.7 |
| City | 2 | 14.3 |
